# Trends and Gaps in Prescribed Burning Research

**DOI:** 10.1007/s00267-025-02119-z

**Published:** 2025-01-31

**Authors:** Luke Gordon, Maldwyn John Evans, Philip Zylstra, David B. Lindenmayer

**Affiliations:** 1 Fenner School of Environment and Society, The Australian National University, Canberra, ACT 2601 Australia; 2 School of Molecular and Life Sciences (MLS), Curtin University, Perth, WA 6102 Australia

**Keywords:** Hazard reduction, Prescribed burn, Ecological burn, Structural topic model, Text analysis, Wildfire

## Abstract

Prescribed burning is a key tool in land management globally used to reduce wildfire risks and achieve ecological, cultural and resource management objectives across both natural and human systems. Despite its widespread application, research on prescribed burning is marked by significant gaps. Subsequently, we posed the following research questions: **(1)** What are the key research topics that define international, peer-reviewed literature on prescribed burning? **(2)** What are the temporal and spatial trends of these topics? **(3)** What are the relationships between the national income of a given country and the trends in research topics? And, **(4)** What are the most salient knowledge gaps in peer-reviewed prescribed burning research, and how can they be addressed? We used structural topic modelling and geoparsing to conduct a detailed text analysis of 7878 peer-reviewed articles on prescribed burning. We revealed that research on prescribed burning is dominated by studies from high-income countries, particularly the United States. This highlights a geographical bias that may skew global understanding and application of prescribed burning practices. Our topic modelling revealed the most prevalent topics to be *Fire Regimes* and *Landscape Biodiversity Management*, whilst topics such as *Air Pollution & Health*, and *Wildfire Risk Management* gained prominence in recent years. Our analysis highlighted a disconnect between forestry-related research and broader landscape management topics. This finding emphasises the need for more interdisciplinary research, and research on the use and effects of prescribed burning in diverse ecosystems and underrepresented regions, particularly in the context of climate change.

## Introduction

Fire is a key ecological process in many terrestrial ecosystems globally (Bowman et al., 2009; Pausas and Keeley, 2021). Repeated, extensive, high-intensity, and high-severity wildfires occur in a wide range of ecosystems worldwide in response to both natural and human-induced drivers (Collins et al., 2021; Levine et al., 2022; Doherty et al., 2024). The frequency of severe wildfires is increasing rapidly (Cunningham et al., 2024). Such fires have prompted widespread discussions on how fire within forests and other vegetation types might be managed to maintain ecosystem integrity (Lindenmayer and Zylstra, 2024), as well as to limit the loss of human life and damage to property and infrastructure (Moritz et al., 2014; Covington and Pyne, 2020). Several kinds of human intervention aimed at reducing fire severity and burnt area are commonly applied, including mechanical thinning (Noss et al., 2006; Kalies and Kent, 2016; but see Taylor et al., 2020) and prescribed burning (Fernandes and Botelho, 2003; Boer et al., 2009; McCaw, 2013; Tolhurst and McCarthy, 2016).

A prescribed burn, also referred to as a ‘controlled burn’ or ‘hazard reduction burn’, involves intentional application of fire to a selected area, under suitable weather conditions, to promote resource or ecological management outcomes (Fernandes and Botelho, 2003; Mason and Lashley, 2021). The objective of prescribed burning is often fire hazard reduction (Kalies and Kent, 2016). However, prescribed burning is also used for other purposes, including to achieve agricultural, ecological, cultural, and forest management objectives (e.g. Lindenmayer et al., 2017; Morgan et al., (2020); Ngwenya et al., 2024).

There is a large, complex, and rapidly expanding research literature on prescribed burning in a range of ecosystems. Exploring research themes within the scientific literature on prescribed burning can provide valuable insights into the main threads of enquiry, the disciplines and subdisciplines in which prescribed burning is studied, how it has developed over time, and in what regions it is studied. This exploration will also highlight potential gaps in knowledge to fill.

Here, we used a combination of modern text-analysis techniques, including topic modelling (Roberts et al., 2014) and geoparsing (D’Ignazio et al., 2014; Westgate et al., 2015; Luiz et al., 2019; Millard et al., 2020; Evans et al., 2021; Andrew et al., 2022), to examine the peer-reviewed literature on prescribed burning. Our aim was to answer the following core research questions: **(1)** What are the key research topics that define international, peer-reviewed literature on prescribed burning? **(2)** What are the temporal and spatial trends of these topics? **(3)** What are the relationships between national income of a given country and the trends in research topics? And, **(4)** What are the most salient knowledge gaps in peer-reviewed prescribed burning research, and how can they be addressed?

Quantifying aspects of the scientific peer-reviewed literature on prescribed burning is crucial. The use of fire in land management can have marked impacts on natural systems – both positive and negative (Kelly et al., 2020). Although there have been some narrative reviews of the literature (e.g. Fernandes and Botelho, 2003; McCaw, 2013) and some location-specific reviews (e.g. Kalies and Yocom Kent, 2016), to the best of our knowledge, this is the first data driven review of the global prescribed burning literature.

## Methods

### Corpus collation

We followed the PRISMA 2020 guidelines in the collation of our corpus (Page et al., 2021) (Fig. S1). On the 14^th^ of August 2023, we conducted title, abstract, and keyword searches in the Scopus and Clarivate Analytics Web of Science Core Collection indexers, using a compiled search term constructed from the authors’ expertise in the area and knowledge of the literature (Table S1). To capture the largest possible range of relevant articles related to prescribed burning, we tested and refined broad search terms over multiple iterations of our literature search (Fig. S1). We used ‘prescribed burning’ and related terms such as ‘hazard reduction’, ‘controlled burn’, and ‘cultural burn’ in conjunction with more specific terms such as ‘fuel’, ‘property’, ‘biodivers*’, and ‘spatial’ (Fig. S1). We did not use limiting search operators such as NOT and SAME. We note that this corpus may not encompass all prescribed burning research as it does not include grey literature on the subject (Mering, 2018) and is limited to those articles accessible in English. However, we believe it is broadly representative of the current peer-reviewed literature on prescribed burning due to the large number of sources we examined from reputable academic databases.

### Inclusion criteria

We combined search results from Web of Science and Scopus and removed duplicates automatically in R (duplicates removed = 4570) (R Core Team 2023). We then manually screened the titles, keywords, and abstracts of each article in our corpus, and removed articles that did not correspond with our inclusion criteria or duplicates missed in our previous screening (duplicates removed = 2197) (Fig. S1). Our inclusion criteria were as follows. We retained those articles that explicitly focused on: **(1)** the use and effectiveness of prescribed burning for landscape management, **(2)** the impacts of prescribed burning on ecological and human systems, **(3)** the relationships between prescribed burning, fuel descriptors and fire severity, and **(4)** how prescribed burning can affect human health. We also excluded articles that were solely literature reviews and included no original research. A total of 7878 articles remained for analysis following screening (Fig. S1).

### Topic modelling

Topic modelling is a machine learning analysis technique used to identify patterns and structures within a large corpus of text. Common probabilistic topic models include latent semantic indexing (Dumais, 1994), latent dirichlet allocation (see Blei et al., 2003; Blei 2012), dynamic topic modelling (Blei, Lafferty 2006), controlled topic modelling (see Blei, Lafferty 2006) and structural topic modelling (STM) (Roberts et al., 2014; Roberts et al., 2019). Topic modelling uses words that co-occur frequently to identify a given number of topics. These co-occurring words represent a meaningful consolidation of language and ideas (Westgate et al., 2015; Evans et al., 2023). For our study, we elected to use STM, as it extends latent dirichlet allocation to allow for the integration of metadata such as publication date and location of study (Roberts et al., 2014; Roberts et al., 2019).

We prepared the corpus for analysis by removing words with fewer than three characters, words that contained foreign characters, punctuation, numbers, and stop words (e.g. the, or, and). We also converted words to their root form (for example, burn = burned, burnt, burning), and removed the most common words ( > 85%) and least common words ( < 1%) which provided limited meaningful information (Westgate et al., 2015). A number of topics must be chosen when fitting an STM to a corpus, and it is best practise to choose a number that isn’t too large or too small, with the aim of specifying a number that is easy to interpret yet captures the highest diversity of topics (Weston et al., 2023). To choose the most appropriate number of topics, we used the ‘searchK’ function from the ‘stm’ package (Roberts et al., 2019) in R (R Core Team 2023) to compare the exclusivity, semantic coherence, held-out likelihood, and residual in models ranging from five to forty topics. After fitting and visualising the most appropriate numbers using this method, we chose 21 as the number of topics for analysis (Westgate et al., 2015; Luiz et al., 2019; Westgate et al., 2020; Evans et al., 2021; Andrew et al., 2022; Evans et al., (2023)). We used spectral initialisation to fit the model, as it has been shown to produce the most reliable results in a shorter timeframe than other methods (Roberts et al., 2016). Following the model fit, we used the top 20 most common words in each topic to assign titles that summarise their content (Westgate et al., 2015) (see Table 1).

**Table 1:** 21 Topics modelled from 7,878 research articles about prescribed burning

Topic #	Topic Title	Brief description	Top 20 words
**1**	Fire Regimes	Prescribed burning research about fire regimes, management, and effects.	fire, prescrib, burn, effect, use, season, manag, sever, nation, regim, park, tool, studi, veget, frequenc, year, object, intens, differ, area
**2**	Biomass & Emissions	Research on the relationship between prescribed burning, biomass, and the emissions these burns produce.	biomass, burn, emiss, carbon, combust, atmospher, aerosol, organ, measur, sourc, gas, sampl, black, residu, matter, particl, factor, composit, chemic, compound
**3**	Soil & Litter	Research into the effects of prescribed burning on soil chemistry and microbial activity.	soil, nitrogen, nutrient, prescrib, organ, properti, fire, effect, burn, microbi, cycl, forest, carbon, miner, avail, phosphorus, ecosystem, studi, activ, chemic
**4**	Sagebrush Steppe Restoration	Research into the effectiveness of prescribed burning as a treatment for sagebrush ecosystem restoration.	artemisia, sagebrush, recoveri, tridentata, stepp, bromus, basin, veget, big, greater, communiti, ecosystem, prescrib, fire, great, treatment, respons, restor, annual, western
**5**	Grasslands & Grazing	Research into the use of prescribed burning for grassland and livestock grazing management.	grassland, prairi, graze, burn, rangeland, restor, state, tallgrass, prescrib, unit, season, product, north, plain, grass, livestock, manag, communiti, respons, cattl
**6**	Air Pollution & Health	Research on the role of prescribed burning in worsening air pollution and human health.	smoke, air, qualiti, health, pollut, impact, exposur, burn, human, particul, wildland, prescrib, matter, environment, monitor, state, fire, diseas, studi, unit
**7**	Pine Forests & Plantations	Research on the use of prescribed burns in pine-conifer forest restoration and plantations.	pine, pinus, stand, longleaf, palustri, ecosystem, restor, prescrib, understori, forest, southeastern, plantat, state, burn, forestri, unit, conifer, regener, treatment, mill
**8**	Climate Change & Carbon	Research into the effects and use of prescribed burning in the context of climate change and carbon sequestration.	chang, climat, ecosystem, carbon, fire, global, forest, increas, wildfir, terrestri, impact, servic, drought, storag, sequestr, regim, climate-chang, can, due, stock
**9**	Forestry	Research into the role of prescribed burns as a forestry tool in the production of timber products.	forest, australia, forestry, harvest, boreal, burn, eucalyptus, site, dri, timber, prescrib, log, wood, eucalypt, effect, debri, fire, product, studi, silvicultur
**10**	Fuels & Fire Behaviour	Prescribed burning research focused on the effects of fuels and flammability characteristics in predicting fire behaviour.	fire, fuel, behaviour, moistur, temperatur, heat, load, rate, behaviour, spread, surfac, characterist, experiment, consumpt, ignit, intens, predict, flame, flammabl, thermal
**11**	Oak Forest Restoration	Research on the use of prescribed burning for the restoration of Oak forests, particularly in North America.	oak, quercus, state, unit, florida, fire, regener, hardwood, eastern, appalachian, speci, prescrib, wetland, USA, central, upland, decidu, north, structur, scrub
**12**	Wildfire Risk Management	Research regarding the use of prescribed burning in the management and reduction of wildfire risk to life and capital.	wildfir, fuel, manag, risk, reduct, fire, hazard, treatment, reduc, econom, increas, public, polici, mitig, cost, assess, forest, wildland, interfac, research
**13**	Hydrology & Erosion	Research into the effects of prescribed burning on hydrological processes and increasing soil erosion due to runoff.	water, mediterranean, eros, wildfir, soil, burn, runoff, hydrolog, prescrib, spain, ash, surfac, effect, sediment, watersh, studi, impact, post-fir, veget, sever
**14**	Remote Sensing Models	Research on the use of remote sensing and geospatial models in understanding the effects and efficacy of prescribed burning.	model, fire, analysi, system, use, data, simul, spatial, develop, inform, method, remot, sens, evalu, weather, predict, base, assess, area, plume
**15**	Tree Mortality	Research into the use of prescribed burning in the management of tree mortality with specific reference to beetle predation on the bark of coniferous trees.	tree, ponderosa, mortal, forest, mountain, beetl, pine, growth, fire, bark, oregon, pinus, coleoptera, northern, pseudotsuga, abi, insect, crown, restor, southwestern
**16**	Plant Ecology	Research studies that concern the relationship between prescribed burning and plant community dynamics.	communiti, disturb, plant, speci, divers, fire, seed, structur, respons, ecolog, ecosystem, composit, dynam, veget, function, rich, interact, success, germin, bank
**17**	Wildlife Ecology	Research on how prescribed burning can be used to manage the ecological dynamics of wildlife.	habitat, popul, bird, select, abund, wildlif, anim, manag, use, speci, declin, mammal, nest, prescrib, surviv, respons, ave, deer, small, effect
**18**	Weed Management	Research concerning the use of prescribed burning in the management of exotic and invasive weeds.	control, invas, plant, speci, grass, nativ, biolog, herbicid, burn, weed, exot, manag, annual, growth, poacea, impact, reduc, veget, can, treatment
**19**	Landscape Biodiversity Management	Research into the effects and efficacy of prescribed burning in the management of biodiversity on the landscape scale.	manag, conserv, land, ecolog, landscap, ecosystem, biodivers, natur, fire, practic, australia, use, environment, resourc, area, south, histori, africa, region, knowledg
**20**	Woodland & Savanna Restoration	Research on how prescribed burning can be used for the restoration of woodlands and savannas, with specific reference to its use in decreasing the cover of North American Juniper woodlands.	woodland, savanna, shrub, woodi, restor, veget, cover, encroach, tree, great, juniperus, texa, shrubland, herbac, state, junip, unit, remov, plain, western
**21**	Western American Conifer Forests	Prescribed burning research that is explicitly about the use of fire in the restoration of Western North American Conifer Forests.	forest, state, unit, california, fire, thin, restor, north, america, western, nevada, sierra, treatment, mix, conif, forestri, structur, coniferophyta, conifer, USA

### Post-hoc Topic Model Analysis

**Fig. 1:**
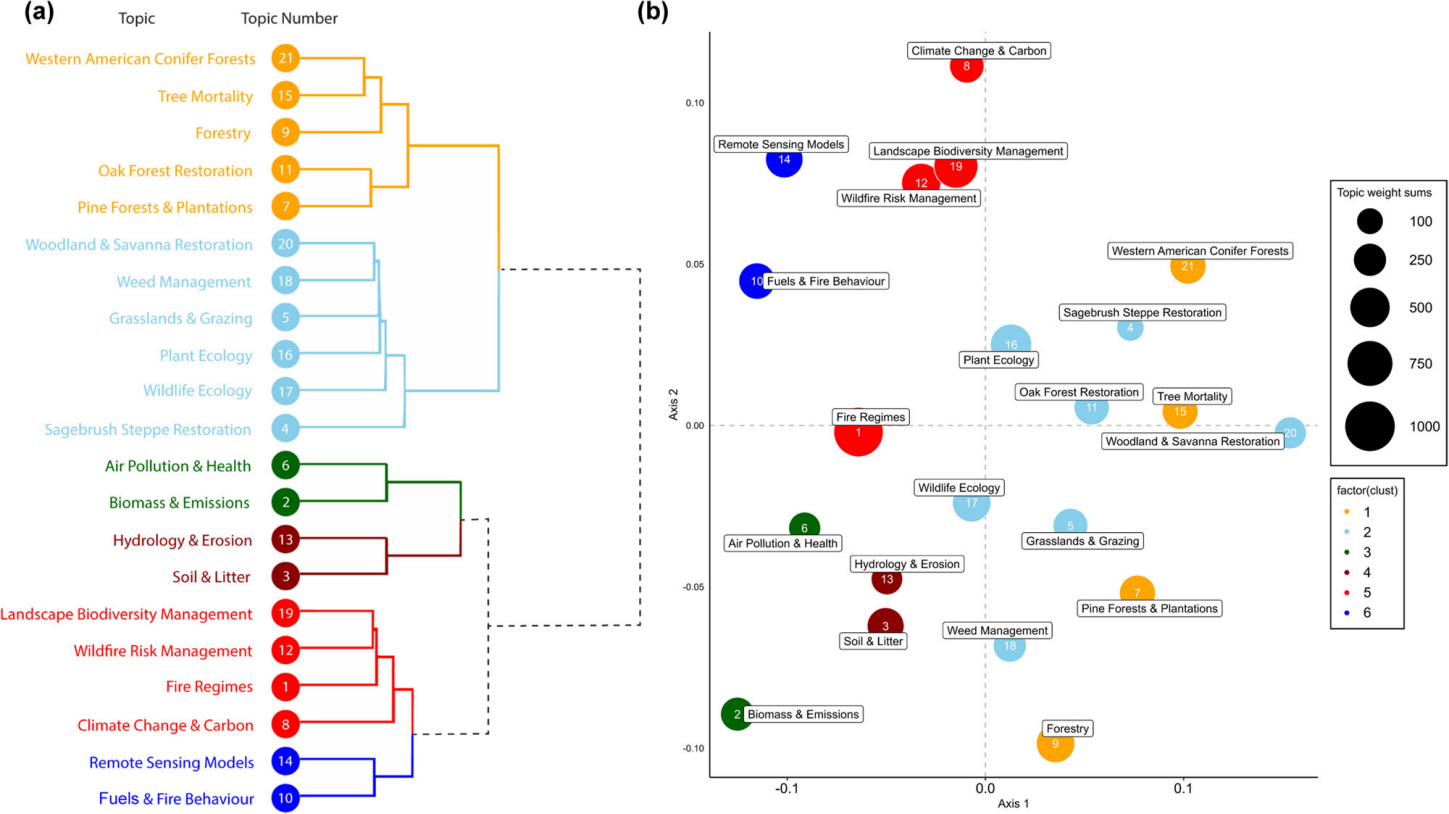
**a** Cluster dendrogram of topics separated by six groups of closely related topics. The black dotted lines represent a virtual link between the separated groups of topics. **b** Non-metric multidimensional scaling ordination plot with colours corresponding to **a** and topic weight sums representing the size of points. Numbers represent the arbitrary topic number given to each topic and this corresponds with those in Table 1

Structural topic models provide a matrix of topic weights for each article that allows for the allocation of articles to the topic with the highest weight. This enables a researcher to undertake further analysis on the results of the topic model, such as comparing topic similarities and differences, popularities over time, and how they are distributed through the corpus (Westgate et al., 2015). To explore topic similarity, we used a hierarchical cluster analysis with Ward’s minimum variance method on the squares of the dissimilarities, based on the model-derived probabilities of the word occurrence matrix (Ward 1963). We then clustered the topics in six groups of closely-associated articles (see Fig. 1a). In addition, we conducted non-metric multidimensional scaling ordination to visually depict similarities and differences between topics (Luiz et al., 2019; Evans et al., 2021) (Fig. 1b). To examine the changing prevalence of topics through time, we fitted a linear regression using the ‘estimateEffect’ function from the STM package (Roberts et al., 2019), specifying year as a continuous predictor variable against the topic weights response variable. We interpreted topics as either hot (more prevalent over time) or cold (less prevalent over time) (Westgate et al., 2015; Evans et al., 2021).

In topic models, topics may reflect moderate associations across many articles (i.e., general topics), and others may reflect strong associations to a small number of articles (i.e., specific topics). To examine this in our analysis, we calculated the mean weight of each topic for the articles where that topic was not the highest-ranked (unselected articles), and then divided this by the mean weight of the articles where the topic was the highest-ranked (selected articles) (Westgate et al. 2015). This resulted in a variable where low values indicated specific topics with distribution of weights biased towards a low number of articles (low weight in unselected articles, high weight in selected articles), and high values indicated general topics where topic weights were distributed more widely across the corpus (high weight in unselected articles, low weight in selected articles) (Westgate et al., 2015; Evans et al., 2023).

### Geoparsing

Geoparsing is the process that allows location names within text to be identified, resolved, and assigned geographical coordinates, usually for the purpose of spatial mapping (Leidner and Lieberman, 2011; D’Ignazio et al., 2014; Millard et al., 2020; Evans et al., (2023); Evans et al., 2023). We used the CLIFF-CLAVIN geoparser (D’Ignazio et al., 2014) to analyse all article abstracts and titles for geographic mentions, which were then subsequently resolved for the most probable physical coordinates (Millard et al., 2020). We ran CLIFF-CLAVIN in Python using a Docker container (Merkel, 2014) hosted on a GitHub repository (Evans et al., 2023). Following this, we categorised the geographic mentions into two groups; ‘country’ (any mention of country or location within it) and ‘locality’ (specific locations within countries) (Evans et al. 2023). We then plotted these country-level and locality-level geographical mentions on global maps in R (R Core Team, 2023) according to each articles’ allocated topic derived from highest topic weight (Fig. 4). To understand the role of national income in determining the location of these studies, we assigned each country an income bracket as defined by the World Bank Gross National Income per capita groups (World Bank Group, 2023), and plotted the total articles published for each bracket in R (R Core Team, 2023) (Fig. S4).

## Results

Our search terms resulted in a corpus of 7878 unique academic articles, with the earliest study in 1950, and the latest study now published in 2024.

### Topics

Our structural topic model identified 21 topics (Table 1) including topics concerned with ecology, forestry, landscape management, biodiversity, soil and litter, erosion, climate change, computational models, air pollution, and wildfires (Table 1, Fig. 1). We recognise that some smaller topics will be missing from this analysis due to limitations in the model, including a lack of sources from prior to 1950, such as those on ‘Indian Burning’. *Fire Regimes* was the largest topic by a considerable margin (topic weight = ~ 948), followed by *Landscape Biodiversity Management* (topic weight = ~ 663), and *Plant Ecology* (topic weight = ~ 551). The least prevalent topic was *Sagebrush Steppe Restoration* (articles = ~ 113), followed by *Hydrology & Erosion* (topic weight = ~ 210), and *Air Pollution & Health* (topic weight = ~ 222) (Fig. S2). Topic clustering and non-metric multidimensional scaling ordination revealed how topics could be divided into six groups ranging from *Forestry and Conifer Forests*, to *Fire Models and Remote Sensing* (Groups 1 to 6 respectively) (Fig. 1). Some topics were neatly clustered with others, such as Groups 4 and 6, whilst others were less so, such as Groups 1 and 2 (Fig. 1). Topic 1 (*Fire Regimes*) was initially somewhat difficult to define as a distinct field of research due to the prevalence of common words co-occurring across many topics.

**Fig. 2:**
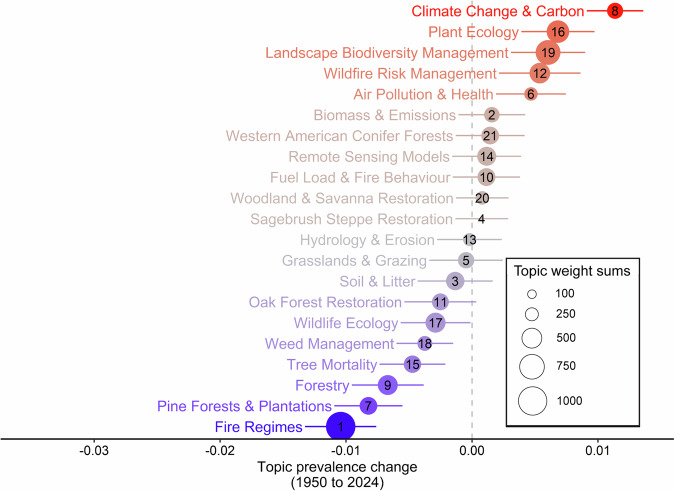
Topic prevalence through time using structural topic modelling and fitting to estimate the linear effect of prevalence between 1950 and the start of 2024. The size of each point represents the sum of topic weights and relative prevalence of the topics across the entire corpus. Those points to the right of the dashed zero-effect line are considered hot topics (red), whereas those to the left are considered cold topics (blue). The error bars running horizontally depict a 95% confidence interval. Numbers represent the arbitrary topic number given to each topic and this corresponds with those in Table 1

The hottest topics (those with increasing prevalence overtime) were *Climate Change & Carbon*, followed in order by *Plant Ecology*, *Landscape Biodiversity Management*, and *Wildfire Risk Management* (Fig. 2). Meanwhile, the coldest topics (those with decreasing prevalence overtime) were *Fire Regimes*, followed by *Pine Forests & Plantations*, *Forestry*, and *Tree Mortality*. Many topics could be deemed as neutral (continuing prevalence overtime) such as *Hydrology and Erosion* (Fig. 2). Eleven topics contained error bars that overlapped a with a neutral topic prevalence of zero, whilst five topics could be considered hot and five others as cold (Fig. 2).

**Fig. 3:**
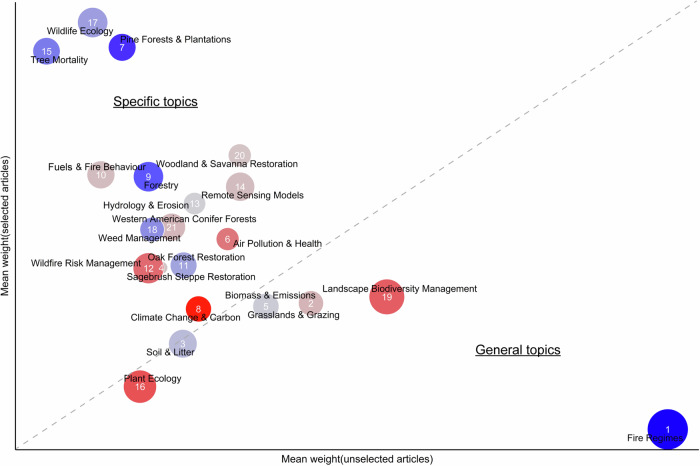
Topic generality and specificity measured as comparison of a topic’s mean weight in articles selected versus unselected to represent that topic. The shade of each topic point represents its topic prevalence through time depicted in Fig. 2. The size of each topic point represents the total number of articles most correlated to that topic as depicted in Fig. 2. Further explanation can be found in the methods section

*Fire Regimes* was the most general topic by a large margin (the topic with the highest weight in unselected articles and lowest weight in selected articles), followed by *Landscape Biodiversity Management* (Fig. 3). The most specific topic was *Wildlife Ecology* (the topic with the lowest weight in unselected articles and highest weight in selected articles), followed closely by *Tree Mortality* and *Pine Forests & Plantations* (Fig. 3).

### Geoparsing

Geoparsing revealed geographical mentions in 4287 of the 7878 articles in the corpus (Fig. S1). The United States recorded the highest number of article mentions (*n* = 2485), approximately five times the number of article mentions than the next highest country, Australia (*n* = 477).

## Discussion

We used text analysis to produce a data-driven overview of peer-reviewed prescribed burning literature available online. The most popular topics across the corpus, such as *Fire Regimes, Landscape Biodiversity Management* and *Plant Ecology*, which were concerned primarily with the interaction between fire and environmental landscapes, were distributed more generally across the corpus than most other topics (Fig. S2, Fig. 3). The topic *Fire Regimes* was found to be the largest, most general, coldest, and widest geographically-spread topic. This suggests that the language used in this broad topic (e.g. prescribed, burn, regime, severity), whilst being common to a large proportion of articles within the corpus, is perhaps declining in popularity as a result of a refinement in disciplinary language over time (Fig. 3). Indeed, this language could be considered more ‘traditional’, than the more specialised language characterising hotter topics, such as *Air Pollution & Health* and *Climate Change & Carbon*.

### Geographic bias and implications for prescribed burning

**Fig. 4:**
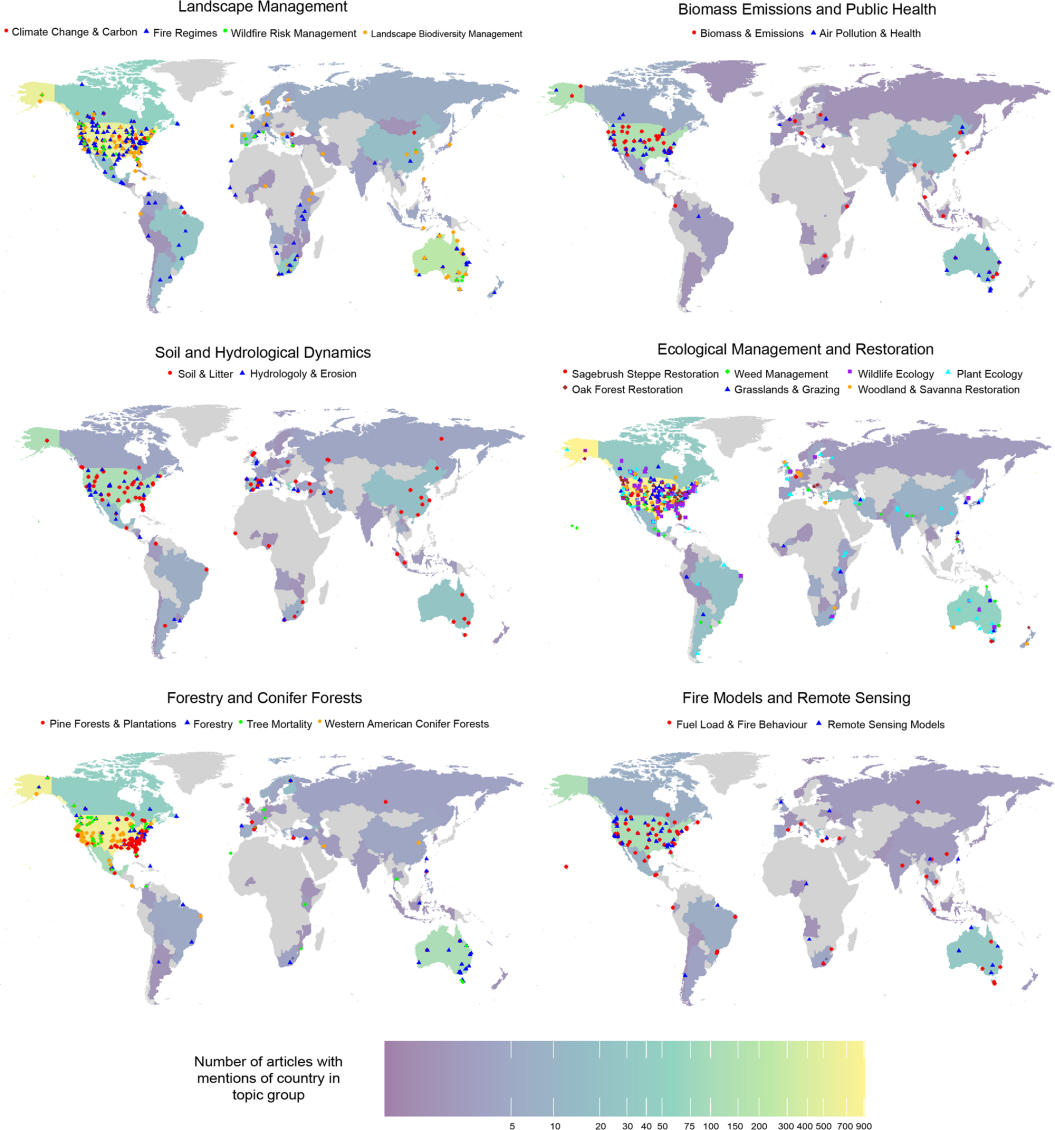
Global maps presenting the unique country mentions (shading of country polygons) and unique locality mentions (points) for articles in their highest ranked topic. Each group represents those closely related topics from the 4287 articles in which a geolocation could be determined. Grey polygons represent no mentions in that country

We identified a distinct geographical bias in the prescribed burning literature towards higher-income countries, especially those in North America. North and Central America comprised ~ 66% of all article mentions resolved by geoparsing, with the United States (U.S) comprising ~ 58%, illustrating how prescribed burning research is dominated by studies in this country (Fig. 4, Fig. S3). Similarly, when accounting for each country’s GNI per capita group as allocated by the World Bank Group (2023), ~ 85% of articles were from high-income countries and ~ 13% were from upper-middle income countries. In contrast, only ~ 2% came from lower-middle income countries, and just ~ 0.8% of articles came from low-income countries (Fig. S4). Not only does this reveal how the location and productivity of economies can influence the prevalence of prescribed burning research, but it also raises the question of how this bias might shape global thinking about, and application of, forest management practices that utilise prescribed burning. For instance, Fernandes and Botelho (2003) provide a widely cited and detailed review of the application and effectiveness of prescribed burning for fuel hazard reduction, but focus on studies only from North America, Australia, and Europe. Similarly, in their literature review on relationships between prescribed fire and wildfire regimes, Hunter and Robles (2020) note that the vast majority of the analyses they studied were located in North America, the Mediterranean, and Australia. Similar geographic biases have been reported in the publications or citations of research in other disciplines (Hamel 2007) such as conservation science (Meijaard et al., 2015; Di Marco et al., 2017), forest science (Boshoff et al., 2024), biodiversity in human-modified landscapes (Trimble and van Aarde, 2012), and nature’s mental health effects (Gallegos-Riofrío et al., 2022). Therefore, a substantial research gap exists for knowledge on the efficacy and effects of prescribed burning treatments in many areas outside of North America, and particularly in lower-income countries. This knowledge gap is highly consequential. The largest study of prescribed burning leverage was conducted in south-east Australian states (Price et al., 2015), and found that even within this range, prescribed burning could have any effect ranging from decreasing wildfire to more often increasing it. If this is even the case in Australian landscapes dominated by eucalypt forest, then appropriate fire management actions in the pine forests of North America are more than likely inappropriate for application in other locations due to varying fire regimes, differing ecosystems and landscape heterogeneity. Whilst our model is likely missing many sources unavailable in English, addressing this knowledge gap is vital, especially given increasing wildfire severity and frequency associated with climate change (Fernandes et al., 2013; Clarke et al., 2019; Duane et al., 2019; Kupfer et al., 2020; Russell-Smith et al., 2020).

The geographical bias in the literature may also permeate the understanding and application of prescribed burning within the different ecosystems of individual countries. ‘Wilderness fire’ or ‘prescribed natural fire’ literature explores how the role of natural fire regimes can shape ecosystems and environments in complex ways, resulting in patterns of landscape heterogeneity (Agee, 2000; Miller, 2014). In the review by Agee (2000), the author identifies four general themes of wilderness fire science at that time: (1) the limits of models and data availability; **(2)** the inherent complexity of fire at a micro and macro scale; **(3)** accounting for fire as a landscape process; and **(4)** the barriers to expanding prescribed wilderness fires. In a subsequent review of wilderness fire science 15 years later, Miller and Aplet (2016) concluded that although much had been learned about fire behaviour and effects across some landscapes and regions, this added to the complexity and uncertainty of fire management decisions. They argued that more studies in differing ecosystems and landscapes were needed to address such complexity at the level of the individual decision maker. Similarly, after the southeastern Australian megafires of 2019–2020, there were increased demands for the use of cultural burning, traditionally used by the First Nations people, as prescribed burning for hazard reduction to prevent high-severity fires (Binskin et al., 2020; Clarke et al., 2022; Mariani et al., 2022). Lindenmayer and Bowd (2023) explained how cultural burning and prescribed burning were markedly different kinds of fire, especially in terms of scale, intensity, heterogeneity and frequency. Moreover, Lindenmayer et al., (2024) explained how in some ecosystems like the tall, wet Mountain Ash (*Eucalyptus regnans*) forests of south-eastern Australia, repeated burning can have perverse effects, leading to ecosystem collapse. In yet other ecosystems, prescribed fire can increase fire severity in the short to medium term (Prober et al., 2016; Zylstra et al., 2022). In other studies, prescribed burns have been found to have negative impacts on biodiversity (Dixon et al., 2018). The fact that our topic modelling process did not differentiate cultural burning as its own distinct topic, suggests that more attention may be required for cultural burning practices in peer-reviewed academia (but see Lindenmayer and Bowd, 2023). Future research must address the conflation of cultural burning with prescribed burning, separately focusing on the effects of both in different ecosystems, regions, and countries – especially those locations where there has been limited research on the subject.

### Landscape Management

Our analysis showed that there were many articles focused on the use of prescribed burning in landscape management. Such studies, however, were absent from many parts of the world and there was a distinct lack of overlap between some topics relevant to the use of prescribed burning in landscape management. *Fire Regimes* was the largest but coldest topic, whilst the *Landscape Biodiversity Management* and *Wildfire Risk Management* topics were found to be large, and the third and fourth most popular respectively (Fig. S1, Fig. 2). This suggests a move away from research on the fire regimes of particular landscapes, to how these fire regimes can be interpreted for the management of landscape biodiversity and wildfire risk. The close relationship between the topics *Landscape Biodiversity Management*, *Wildfire Risk Management*, and *Climate Change & Carbon*, indicates how these concepts are often treated holistically in literature concerning the use of prescribed burning for landscape management (Fig. 1) (Fernandes et al., 2013; Duane et al., 2019; Morgan et al., 2020). In contrast, the distant relationship between these topics and those such as *Forestry*, *Pine Forests & Plantations*, *Biomass & Emissions*, and *Weed Management*, suggests these concepts are often not analysed in conjunction with regards to the use of prescribed burning in the management of various landscapes. Furthermore, regardless of the fact that the topics *Fire Regimes, Landscape Biodiversity Management* and *Wildfire Risk Management* were large in comparison with others in this analysis, they still suffered from an innate geographical bias towards higher-income countries (Fig. 4).

### Disconnect Between Disciplines

We found that some prescribed burning topics were not as closely related to landscape management as may have been expected, suggesting a gap in the research between these topics. For example, *Forestry* was found to be the furthest away from *Climate Change & Carbon, Landscape Biodiversity Management, Remote Sensing Models* and *Wildfire Risk Management* (Fig. 1). In addition, forestry-related topics clustered separately from topics related to ecology or biodiversity (Fig. 1). This finding suggests that the majority of prescribed burning articles concerning forestry do not include reference to other priorities in landscape management, such as biodiversity conservation and wildfire risk. *Forestry* and *Pine Forests & Plantations* may also be unrelated to landscape management topics due to their ranks as the second and third least popular topics respectively (Fig. 2), and relatively specific content (Fig. 3), reflecting a lack of interdisciplinarity between these topics. It is also worth noting that whilst prescribed burning studies on *Landscape Biodiversity Management* and *Plant Ecology* are increasing in frequency, those studies on *Wildlife Ecology* are generally decreasing in popularity (Fig. 2). Whereas understanding the ecological effects of prescribed burning on plant communities is important (as they represent the first trophic level (Ebeling et al., 2018)), it is also critical to understand how different applications of prescribed fire can impact wildlife, both positively and negatively (Pastro et al., 2011; Toledo et al., 2012; Dixon et al., 2018), before making fire management decisions. Subsequently, although prescribed burning articles on landscape management often consider various topics together and are common, we recommend that more interdisciplinary studies be conducted into the effects of prescribed burning in areas designated for native forestry and plantation forestry. We also recommend that more studies be conducted on the effects and efficacy of prescribed burning in the management of wildlife within various landscape contexts.

### Climate Change

Climate change is a pressing matter of concern with regards to the efficacy and effects of prescribed burning for resource management, biodiversity conservation, carbon emissions and hazard reduction outcomes (for example see Penman et al., 2011; Fernandes et al., 2013; Clarke et al., 2019). A recent study by Cunningham et al., (2024) revealed that the frequency of extreme fire events ( ≥ 99.99th percentile) increased by 2.2-fold between 2003 and 2023, with the last seven years including the six most extreme fire events. Our results on prescribed burning showed that *Climate Change & Carbon* was the hottest topic by a significant margin (Fig. 2). Similarly, the close clustering (based on ordination) of this topic with topics such as *Wildfire Risk Management*, *Landscape Biodiversity Management*, and *Remote Sensing Models* indicates that these subjects are often studied together when examining the use of prescribed fire and climate change (for example, see Penman et al., 2011; Duane et al., 2019; Kupfer et al., 2020). However, Climate Change & Carbon was on the lower end of total allocated articles (Fig. S2), and there was a distinct lack of climate change studies linked to specific geographical location. We found that ~ 2/3 (66%) of climate change studies identified in the corpus did not have country or locality level resolved through geoparsing (Fig. 4). This suggests that it is more common for prescribed burning academic articles to study climate change as a global or conceptual phenomenon, rather than in relation to specific ecosystems or landscapes. In addition, some topics (*Air Pollution & Health*, *Biomass & Emissions* and *Forestry*) that would be assumed to be closely related to the study of prescribed burning and climate change, were found to be relatively distant in ordination space (Fig. 1). This finding could be a product of the fact that these topics are characterised by a small number of allocated articles (Fig. S2), or because all three topics were relatively general rather than specific in their content (Fig. 3). It is also possible, however, that there is a gap between the research for each topic. Subsequently, although research into the relationship between climate change and prescribed burning is quickly expanding, we recommend that more research should be focussed on the application of prescribed burning for management outcomes in specific landscapes and under changing climatic conditions.

### Air Pollution and Human Health

Increasing wildfire severity, intensity, and frequency in some landscapes has resulted in prescribed burning becoming a more common practice in an attempt to reduce fuel loads and decrease wildfire risk. However, increased use of prescribed burning produces more woodsmoke, thereby degrading air quality and negatively affecting human health (Wain et al., 2008; Haikerwal et al., 2015; Jaffe et al., 2020). Burning biomass produces fine particulate matter (PM_2.5_ aerodynamic diameter < 2.5 µm), which is harmful to human health (Reisen and Brown, 2006; Naeher et al., 2007; Haikerwal et al., 2015), and due to the atmospheric conditions required for prescribed burning, the impact on human health is much greater per hectare (Borchers-Arriagada et al., 2021), resulting in substantially increased levels of morbidity and mortality (Borchers-Arriagada et al., 2020; Johnston et al., 2014; Reisen and Brown, 2006). Consequently, there is a conflict between the impetus to increase the use of prescribed burning for hazard reduction and resource management outcomes, and the increased hazard that it causes through air pollution (Altangerel and Kull 2013, Jaffe et al., 2020, Jones et al., 2022). Our study suggests there has been a relatively recent surge in research on the relationship between prescribed burning and *Air Pollution & Health*, including research articles on the difference in smoke production between wildfires and prescribed burns (Williamson, et al., 2012, Navarro, et al., 2018). The topic was the fifth most popular at the time of analysis (Fig. 2), but the third smallest (Fig. S2). It is likely that this has coincided with the growth of prescribed burning literature on climate change, due to increased calls for the use of prescribed burning to reduce wildfire hazard and carbon emissions (Narayan et al., 2007; Morgan et al., (2020); Russell-Smith et al., 2020; Pacheco and Claro, 2021), and a small number of researchers responding to hitherto ignored health statistics (see Johnston et al., 2013; Johnston et al., 2014 for example). *Air Pollution & Health* is closely related to *Biomass & Emissions* and *Fire Regimes* (Table 1), reflecting the prevalence of prescribed burning articles associated with this topic that quantify particulate biomass emissions (see Lee et al., 2005; Zhang et al., 2013; Holder et al., 2017 for example). The topic also suffers from a distinct bias in geographical spread towards the U.S and Australia (Fig. 4). Subsequently, we recommend that more research should be directed towards understanding the relationship between prescribed burning and harmful air pollution in varying contexts, both geographically and in relation to future climate scenarios. In addition, we recommend more research on resolving the conflicts between prescribed burning, public health, and resource management outcomes.

### Effectiveness and Risk Management

The topics *Fuels and Fire Behaviour* and *Remote Sensing Models* have either direct or indirect bearing on several of the other topics, as they address the underlying premise that burning for fuel reduction reduces fire frequency or severity. Findings of these studies were at times contrasting or ambiguous, even for the same vegetation communities. For example, one study showed that areas of Ponderosa Pine (*Pinus ponderosa*), where fire had been suppressed, had a greater density of saplings capable of acting as ladder fuels and facilitating crown fire (Battaglia et al., 2018). In contrast, a separate study of long-unburnt Ponderosa Pine reported it to be open and free of ladder fuels (Madany and West, 1983). It appears that other forms of disturbance such as logging or grazing caused the underlying difference between these findings, but in other cases, studies contradicted each other due to statistical issues. One analysis of the efficacy of prescribed burning in mitigating house loss from wildfire found a small protective effect when analysing a partial dataset (Gibbons et al., 2012), but another analysis of the full dataset found that prescribed burning increased the likelihood of both crown fire and house loss (Price and Bradstock, 2013). Lindenmayer and Zylstra (2024) found that studies could produce opposite findings depending on whether or not they captured the long-term effects of treatments. It was beyond the scope of this study to fully interrogate all conclusions, but our study identified a moderate to low level of attention in these topics (Fig. S1), with no significant trend over time (Fig. 2). The topic *Wildfire Risk Management* encompasses the application of the underlying theory to decision making, and it is worth noting that this topic received more attention (Fig. S1) and was the fourth hottest topic. This increasing focus on model application despite conflicting and consistently modest levels of model validation suggests a degree of confidence in the underpinning theory that may not yet be justified.

### Limitations

Although this study presents a detailed summation of prescribed burning research, it is not a perfect depiction and we acknowledge several limitations. First, in the construction of our corpus, we limited our focus to those articles on: **(1)** the use and effectiveness of prescribed burning for management actions, **(2)** its impacts on ecological and human systems, **(3)** the role of fuels and fire severity, and, **(4)** how prescribed burning can affect human health. We also targeted articles that were: **(5)** likely to contain original research. Whilst our relatively broad focus allowed for the collection of many relevant articles, some aspects of prescribed burning literature were not fully captured by our search term, such as those studies concerned with the use of prescribed fire in agriculture-dominated areas. Our study deliberately excluded ‘grey literature’, that is, literature which has been produced or published outside of traditional academic publishing, requiring peer-review as a minimum standard for quality control purposes. This includes reports, government documents and working documents that have not undergone strong scrutiny.

Second, the text-based analysis in topic modelling is often described as allowing the data to ‘speak for itself,’ with topics emerging based on word co-occurrences (Blei et al., 2003). However, this process still involves several subjective decisions and assumptions. For instance, the choice of search terms can significantly impact the results, as different researchers might select terms based on their perspectives, resulting in different outcomes. Additionally, after experimenting with various models and conducting comparative tests, we settled on analysing 21 topics, as we considered the topics produced by this number to be the most comprehensible. Another set of authors may have decided to specify a different number of topics to be the most appropriate. The process of naming these topics also involved subjective interpretation, influenced by our personal experiences and areas of expertise.

Last, we included only those articles available in English for use in the text-based analysis. Subsequently, it may present a perspective and collection of research skewed towards a Eurocentric view of the effects and efficacy of prescribed burning. This is particularly relevant given there are cultural differences in the values, perceptions, and application of prescribed burning across cultures, especially between Eurocentric and Indigenous views (Roos et al., 2021; Rodrigues et al., 2022). Due to the inherent heterogeneity of fire within varied landscapes and the diversity of prescribed burning understanding to match, non-English sources could provide a valuable insight into the field of research. It is likely there are numerous studies on the effects and efficacy of prescribed burning published in other languages, and could not be included in this analysis.

## Conclusions

We have provided an overview of the different topics in the prescribed burning research literature. There is a substantial research gap in the efficacy and effects of prescribed burning treatments in many areas outside of North America, and particularly in lower-income countries. More research needs to be directed toward understanding the effects and efficacy of prescribed burning in different ecosystems, regions, and countries, and especially those locations where there has been limited research on the subject. Similarly, we recommend that more research be focussed on the application of prescribed burning for management outcomes in landscapes under future climate change. More broadly, we note that the rapidly growing emphasis on the application of prescribed burning in risk management outweighs the modest and contested body of evidence forming the foundations of the practice, and suggest that studies account for efficacy over the full period affected by the treatment. We also recommend that research into the use of prescribed burning in forestry take a more interdisciplinary approach, and that more studies be conducted on the effects and efficacy of prescribed burning in the management of wildlife within various landscape contexts. Similarly, there must be a separate focus on the effects of both cultural burning and prescribed burning in different ecosystems, regions, and countries as to avoid conflation of these concepts (Lindenmayer and Bowd, 2023). Lastly, we recommend that more research should be focused on resolving the conflicts between prescribed burning, public health, and resource management outcomes.

## Supplementary information

Supplementary information

## Data Availability

The data and code used for this article will be made publicly available and linked here using a data repository such as Data Dryad should the article be accepted.
