# Supplementary material for: Trends and Gaps in Prescribed Burning Research
Source: Environ Manage. 2025 Jan 31;75(4):746–60. doi: 10.1007/s00267-025-02119-z (PMC11965264; doi:10.1007/s00267-025-02119-z)
Supplement: Supplementary file 1 — Supplementary information [file 267_2025_2119_MOESM1_ESM.docx]

***Environmental Management***

*Research Paper*

**SUPPLEMENTARY INFORMATION**

**Table S1.** Boolean expressions used to discover articles associated with prescribed burning. The OR operator is used in conjunction with the AND operator to expand the possible articles that could be obtained by the search. TS represents a search for the terms provided in the title, abstract and keywords of articles returned by the Web of Science Core Collection database. TITLE-ABS-KEY achieves the same objective within the search term for the Scopus database. Note that the Scopus database allows for a search based off subject areas (SUBJAREA), but this was not used in the Web of Science Core Collection database due to apparent issues with its subject categories at the time of the database search.

| **Database** | **Search term with collective Boolean operators** |
| --- | --- |
| Web of Science | TS = ((“prescribed burn*” OR “prescribed fire*” OR “hazard reduction*” OR “controlled burn*” OR “ecological burn*” OR “planned burn*” OR “cultural burn*”) AND (“fuel reduction*” OR “fuel treatment*” OR “fuel management*” OR “time since fire*” OR effect* OR severity* OR flammab* OR risk* OR intensity OR fire* OR fuel* OR property OR spatial* OR biodivers* OR conserv* OR forest* OR health*)) |
| Scopus | ( TITLE-ABS-KEY ("prescribed burn*" OR "prescribed fire*" OR "hazard reduction*" OR "controlled burn*" OR "Ecological burn*" OR "planned burn*" OR “cultural burn*”) AND TITLE-ABS-KEY ( "fuel reduction*" OR "fuel treatment*" OR "fuel management*" OR "time since fire*" OR effect* OR severity* OR flammab* OR risk* OR intensity OR fire* OR fuel* OR property OR spatial* OR biodivers* OR conserv* OR forest* OR health*) ) AND ( LIMIT-TO ( SUBJAREA , "ENVI" ) OR LIMIT-TO ( SUBJAREA , "EART" ) OR LIMIT-TO ( SUBJAREA , "SOCI" ) OR LIMIT-TO ( SUBJAREA , "AGRI" ) OR LIMIT-TO ( SUBJAREA , "COMP" ) OR LIMIT-TO ( SUBJAREA , "HEAL" ) OR LIMIT-TO ( SUBJAREA , "BIOC" ) ) |

**Identification of studies via databases and registers**

Records removed *before screening*:

- Non-English language articles were either not picked up in initial searches, or were removed as they cannot work with the text-based topic modelling.
- Results of initial corpus check for obviously incorrect articles. Changes and additions to the search term were implemented to remove these articles

Records identified from:

Web of Science (n = 7,426)

Scopus (n = 7,209)

**Identification**

Inclusion criteria:

Those articles that explicitly focused on (1) the use and effectiveness of prescribed burning for management actions, (2) it’s impacts on ecological and human systems, (3) the role of fuels and fire severity, (4) how prescribed burning can affect human health, and (5) were likely to contain original research.

Duplicate records excluded automatically using code in R

(n = 4,570)

Records excluded after manually screening

(n = 2,197)

**Screening**

Some articles did not have a geolocation identifiable in their title or abstract and so could not be included in the geoparsing analysis.

Articles included in the review

(n = 7,878)

Articles that could be identified for geoparsing analysis

(n = 4,287)

**Included**

**Figure S1.** PRISMA 2020 flow diagram illustrating the process of corpus article collation (Page et al, 2021).


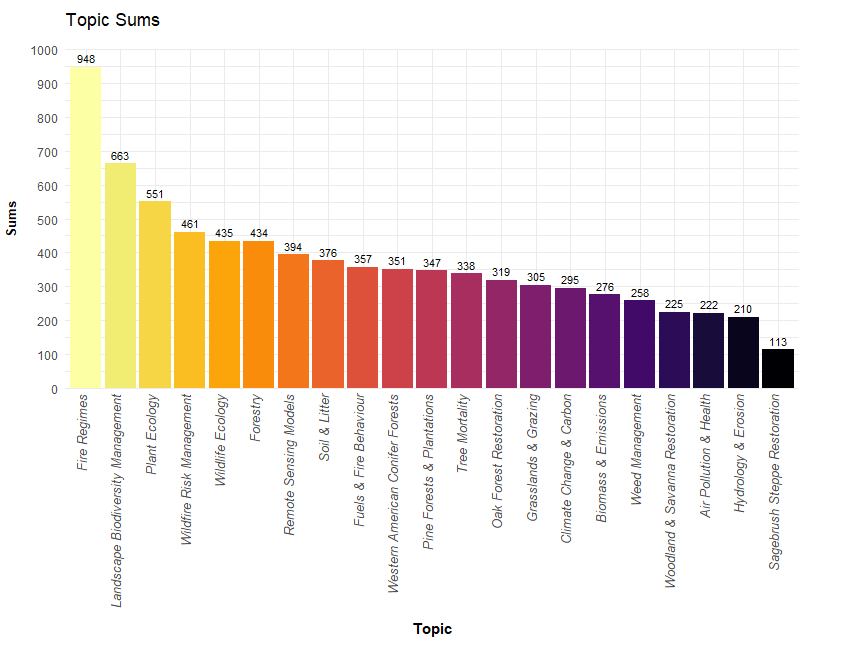
**Figure S2.** Rounded topic weight sums of 7,878 articles correlated to the 21 topics **

Figure S3.** The top 24 countries of study in 4,287 academic articles about prescribed burning using geoparsing. The ‘ggflags’ package (Auguie, 2021) was used in R (R Core Team 2023) in order to plot the circular flags, which originate from EmojiOne (CC-BY-4.0 licence, Brad Erickson).


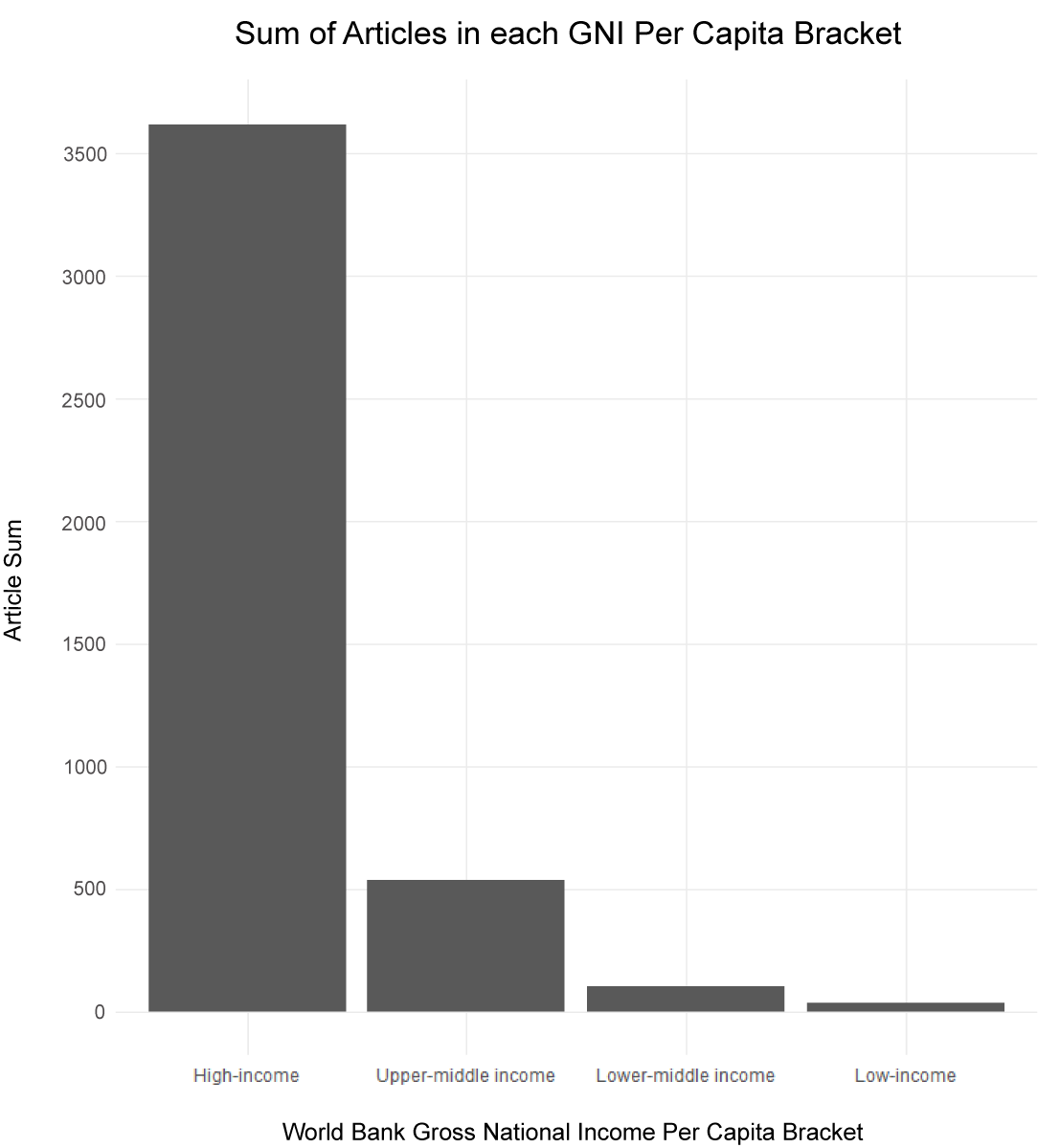


**Figure S4.** The sum of articles in each World Bank GNI per capita bracket resolved with a country reference following geoparsing in R (R Core Team 2023).

**SUPPLEMENTARY INFORMATION REFERENCES**

Auguie, B. (2021). *ggflags: Plot flags of the world in ggplot2.* (GitHub. https://github.com/jimjam-slam/ggflags

Fellows, I. (2018). *wordcloud: Word Clouds. R package* (https://doi.org/https://cran.r-project.org/web/packages/wordcloud/index.html

Page, M. J., McKenzie, J. E., Bossuyt, P. M., Boutron, I., Hoffmann, T. C., Mulrow, C. D., Shamseer, L., Tetzlaff, J. M., & Moher, D. (2021). Updating guidance for reporting systematic reviews: development of the PRISMA 2020 statement. *Journal of clinical epidemiology*, *134*, 103-112. https://doi.org/https://doi.org/10.1016/j.jclinepi.2021.02.003

R Core Team. (2023). *R: A language and environment for statistical computing*. In R Foundation for Statistical Computing. URL https://www.R-project.org/

Roberts, M. E., Stewart, B. M., & Tingley, D. (2019). Stm: An R package for structural topic models. *Journal of statistical software*, *91*, 1-40. https://doi.org/https://doi.org/10.18637/jss.v091.i02
